# Prevalence of Specific Mood Profile Clusters among Elite and Youth Athletes at a Brazilian Sports Club

**DOI:** 10.3390/sports12070195

**Published:** 2024-07-18

**Authors:** Izabel Cristina Provenza de Miranda Rohlfs, Franco Noce, Carolina Wilke, Victoria R. Terry, Renée L. Parsons-Smith, Peter C. Terry

**Affiliations:** 1 School of Psychology and Wellbeing, University of Southern Queensland, Toowoomba 4350, Australia; renee.parsons-smith@boundlesslearning.com (R.L.P.-S.); peter.terry@unisq.edu.au (P.C.T.); 2 Unified Center for the Identification and Development of Performance Athletes (CUIDAR), Clube de Regatas do Flamengo, Rio de Janeiro 22430-041, Brazil; 3 School of Physical Education, Physiotherapy and Occupational Therapy, Federal University of Minas Gerais, Belo Horizonte 31270-901, Brazil; fnoce@hotmail.com (F.N.); carolina.wilke@stmarys.ac.uk (C.W.); 4 Faculty of Sport, Technology and Health Sciences, St. Mary’s University, London TW1 4SX, UK; 5 Centre for Health Research, University of Southern Queensland, Toowoomba 4350, Australia; victoria.terry@unisq.edu.au; 6 School of Nursing and Midwifery, University of Southern Queensland, Toowoomba 4350, Australia

**Keywords:** Brazil, management, wellbeing, emotion, performance, elite sport, youth sport, mood profile, elite athletes, young athletes

## Abstract

Those responsible for elite and youth athletes are increasingly aware of the need to balance the quest for superior performance with the need to protect the physical and psychological wellbeing of athletes. As a result, regular assessment of risks to mental health is a common feature in sports organisations. In the present study, the Brazil Mood Scale (BRAMS) was administered to 898 athletes (387 female, 511 male, age range: 12–44 years) at a leading sports club in Rio de Janeiro using either “past week” or “right now” response timeframes. Using seeded k-means cluster analysis, six distinct mood profile clusters were identified, referred to as the iceberg, surface, submerged, shark fin, inverse iceberg, and inverse Everest profiles. The latter three profiles, which are associated with varying degrees of increased risk to mental health, were reported by 238 athletes (26.5%). The prevalence of these three mood clusters varied according to the response timeframe (past week > right now) and the sex of the athletes (female > male). The prevalence of the iceberg profile varied by athlete sex (male > female), and age (12–17 years > 18+ years). Findings supported use of the BRAMS as a screening tool for the risk of psychological issues among athletes in Brazilian sports organisations.

## 1. Introduction

There is a growing awareness in organisations responsible for developing elite athletes, of the need to balance the intense demands of high-performance sports against the preservation of athlete wellbeing, in particular their physical and mental health [1,2]. In Brazil, sport is seen as a vehicle for driving positive social transformation [3], which has been particularly evident since the so-called decade of mega-events that included hosting the 2014 Football World Cup and the 2016 Summer Olympic Games [4]. High-performance athlete development in Brazil often sits within the confines of large multi-sport clubs [5], sometimes with more than 1000 athletes in intensive training, ranging from talented children through to elite performers on the world stage. Such talent is drawn both from socially disadvantaged groups, living in what are commonly referred to as “favelas”, through to the children of affluent families.

An important feature in the evolution of high-performance sports environments is the proliferation of specialized support personnel, such as sports scientists, strength and conditioning experts, dieticians, sports psychologists, and sports medicine specialists [6,7]. In a contemporary high-performance environment, management strategies should attempt to maximize benefits and minimize harm [1,8]. Athletes involved in high-level sports experience a range of stressors that increase their risk of mental ill-health, including physical injuries, performance slumps, a tendency towards maladaptive perfectionism, and extreme competition to be selected for teams [2,8]. The risk of mental health issues is further increased by general stressors that include adverse life events, financial uncertainty, low self-esteem, social media abuse, or inadequate social support [9]. In a systematic review, Gouttebarg et al. [10] estimated that about one-third of high-level athletes reported some symptoms of mental ill-health, especially depression and anxiety.

Mood profiling is a longstanding method of screening athlete individuals and groups for mental ill-health risk and prediction of performance [11,12,13] and is used widely in Brazil [14]. Moods are reflective of how we interact with people and the environment around us, and can therefore influence mental health, life in general, and sports performance [12]. Beedie et al. [15] proposed that the ability to recognize and manage emotions and moods is fundamental to the sustainability of mental health, quality of life, and performance. Moods have been conceptualized as a collection of low-intensity internal feelings that often lack identifiable triggering events and are typically less intense and tend to persist longer than emotions, which can be seen as intense and conscious feelings in response to an external event or cue [16,17]. Moods are typically viewed as having both a valence dimension from positive to negative and an arousal dimension from activation to deactivation [18]. 

Moods are frequently assessed using self-report scales such as the Profile of Mood States (POMS) [19] or one of its derivatives, such as the Brunel Mood Scale (BRUMS) [20]. Mood profiling involves plotting raw scores on the POMS or BRUMS against test norms and presenting the standardized scores graphically. The resultant visual profiles can be used to identify common patterns of mood responses and to assess the relationships between mood, performance, and psychological wellbeing [11,12,13,14]. Rohlfs and associates developed and validated a Brazilian Portuguese version of the BRUMS, referred to as the Brazil Mood Scale (BRAMS) [14,21], which facilitated further investigations of mood-related topics in Brazil. Since then, mood profiling has been used in Brazil in numerous published studies, covering diverse contexts, including prevention of overtraining syndrome [21,22,23], evaluation of cardiac rehabilitation patients [24,25], monitoring performance and moods of youth athletes [26,27], monitoring moods among members of the national basketball, futsal, gymnastics, jiu-jitsu, judo, sailing, soccer, swimming, tennis and volleyball teams [28,29,30,31,32], monitoring training load and recovery in elite athletes [33,34], assessing moods of women with fibromyalgia [35,36,37], associating sleep quality with mood responses in elite athletes [38,39,40], and comparing lifestyle parameters and mood states of active and sedentary people [41,42]. 

Recently, the measurement model of the BRAMS was re-evaluated, with support found for both a retrospective response timeframe, “how have you been feeling over the past week including today”, and an immediate response timeframe, “how are you feeling right now” [43]. Retrospective measures are proposed to be extremely useful in sports [13,44], as they can identify if mood has deteriorated due to heavy training loads and/or other aspects of sports participation, helping to inform strategies used by coaches, in partnership with multidisciplinary support teams, to avoid injuries, overtraining, and mental ill-health, among other conditions. Therefore, the BRAMS, using either a retrospective or immediate response timeframe, can be considered to be an appropriate tool for research among Brazilian populations, in our case to investigate the prevalence of particular mood profiles proposed in the extant literature. The profiles in question are referred to as the iceberg, inverse iceberg, inverse Everest, surface, submerged, and shark fin profiles [45,46].

**Figure 1:**
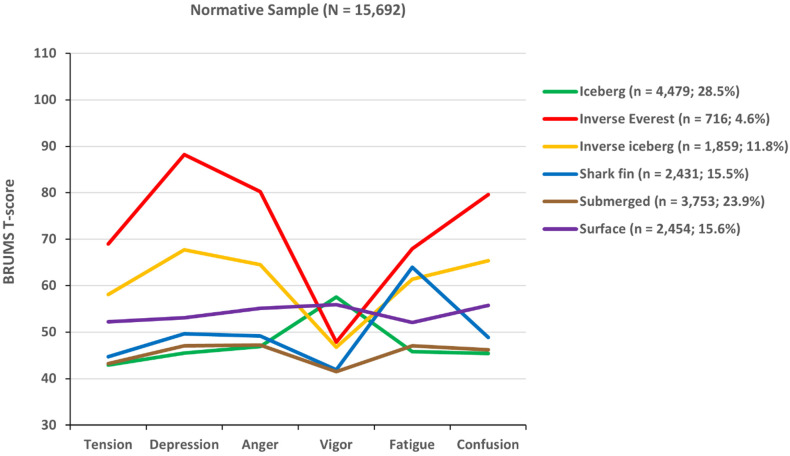
Graphical representation of six mood profile clusters based on 15,692 participants [46].

A graphical representation of the six mood profile clusters is shown in Figure 1, based on a normative sample of 15,692 participants [46]. The iceberg profile [12] (green line) was named as such because, like a real iceberg, most of the profile is below the metaphorical waterline (the mean standardized score of 50), with just the score for vigor protruding above the surface. The iceberg profile has been shown to associate with positive mental health [12] and good athletic performance [11]. Conversely, the inverse iceberg profile (yellow line) shows a below-average score for vigor, combined with above-average scores for tension, depression, anger, fatigue, and confusion. The inverse iceberg profile is typically considered to represent a negative profile that may debilitate performance and represent a risk to mental wellbeing [12,13,47]. The inverse Everest profile (red line) reflects high levels of tension and fatigue, combined with very high levels of depression, anger, and confusion, and low vigor. Such a negative profile is likely to impede performance efforts and is associated with an increased risk of psychological disorders [46]. The shark fin profile (blue line) combines a very high fatigue score with below-average scores for tension, depression, anger, vigor, and confusion, which has been shown to be associated with the risk of athletic injury [48]. The surface profile (purple line), wherein scores on all mood dimensions are close to the mean standardized score of 50, can be seen to represent an average mood [12,46]. Finally, the submerged profile (brown line) has below-average scores on all mood dimensions, which is reflective of a low risk of mental ill-health. It has been suggested that the submerged profile may be beneficial to performance in sports such as shooting and archery, which require performers to remain calm and unemotional [46]. 

The usefulness of measures suitable for screening large numbers of athletes, particularly at the elite level, is well established [1,49], and the identification of mood profiles associated with an elevated risk of psychopathology, including conditions such as chronic fatigue, overtraining, post-traumatic stress disorder, eating disorders, depression, and anxiety, is of great importance, as they can signal the need for follow-up by a mental health professional [46,50]. On a more positive note, coaches, support staff, and the athletes themselves can use evidence-based mood enhancement strategies, such as relaxation techniques, listening to music, or visualization to generate desired mood profiles prior to competitive situations [44,51,52]. The six mood profiles in Figure 1 have been reproduced in several different language and cultural contexts, including Brazilian [53], Chinese [54], English [45,46], Italian [55], Lithuanian [56], Singaporean [57], and most recently, a Greek context [58]. However, to date, they have not been identified in any population using a retrospective “past week” response timeframe. 

Therefore, our first aim was to investigate if the six mood profile clusters identified in the literature [45,46,53,54,55,56,57,58] were also evident among a sample of youth and elite athletes from a sports club based in Rio de Janeiro, Brazil, using both “right now” and past week” response timeframes. It was hypothesized (H1) that the six clusters would be identifiable using both response timeframes. Our second aim, given existing evidence of differences in moods according to the sex and age of participants [59,60,61], was to compare the prevalence of specific mood profile clusters by male/female and youth/adult groups. It was hypothesized (H2) that differences in prevalence would emerge for both sex and age. Finally, evidence showing a link between social vulnerability and mental ill-health [62,63] precipitated a comparison of mood profile clusters between the socially vulnerable and not vulnerable groups. Given evidence of a high prevalence of mental health issues among those living in Brazilian “favelas” [64], it was hypothesized (H3) that negative mood profiles would be more prevalent among socially vulnerable participants.

## 2. Materials and Methods

### 2.1. Participants

A total of 898 athletes from a sports club in Rio de Janeiro, Brazil, participated. The participants were randomly allocated to groups based on the response timeframe used in the assessment of mood (i.e., “right now” or “past week”). One group of 481 athletes (female = 199, male = 282) completed the BRAMS using the “right now” response timeframe. A second group of 417 athletes (female = 188, male = 229) completed the BRAMS using the “past week” response timeframe. 

Comprehensive details of the characteristics of the participants are shown in Table 1. The participants were all athletes affiliated with the Unified Center for the Identification and Development of Performance Athletes (Centro Unificado de Identificação e Desenvolvimento do Atleta de Rendimento (CUIDAR, which is Portuguese for “care”). The CUIDAR initiative is a multidisciplinary program of health professionals who work with coaching and management staff in an integrated way to improve athlete performance while safeguarding athlete’s physical and mental wellbeing. The CUIDAR team includes health professionals from psychology, nutrition, strength and conditioning, massage therapy, medicine, nursing, physiotherapy, social services, and sports science who collectively have responsibility for over 1000 youth and elite athletes. 

Approximately 50% of athletes involved in the CUIDAR program live in areas associated with social vulnerability. The Brazilian Ministry of Health considers an individual to be in a situation of social vulnerability based on their socioeconomic status, exposure to risks (including violence, discrimination, and environmental hazards), adverse health conditions, and membership of specific marginalized groups. Socioeconomic factors such as poverty, lack of access to essential services, and financial insecurity are common indicators of social vulnerability, which in turn increases the risk of mental ill-health [64]. 

### 2.2. Measurement of Mood

The Brazil Mood Scale (BRAMS) [14,43] was used to assess mood. The BRAMS has 24 items, including six subscales, referred to as confusão (confusion), depressão (depression), fadiga (fatigue), raiva (anger), tensão (tension), and vigor (vigor). Participants indicated either how they were feeling “right now” or how they had been feeling over the “past week” by responding to a 5-point scale, where 0 = nada (not at all), 1 = um pouco (a little), 2 = moderadamente (moderately), 3 = bastante (quite a bit), and 4 = extremamente (extremely). Responses for each subscale were summed to give six subscale scores in the range of 0-16. The BRAMS measure has shown adequate concurrent validity, internal consistency, and factorial validity during validation studies [14,43,53]. It is important to emphasize that the BRAMS is not a diagnostic tool, and assesses only six dimensions of mood, including an index of depressed mood. It does not assess clinical depression [20,65]. All subscales of the BRAMS showed reliability (alpha) coefficients exceeding the threshold of acceptability (α = 0.70) in the original validation study [14]. In the present study, alpha coefficients ranged from 0.72 (tension) to 0.87 (anger) for the “right now” response timeframe and from 0.75 (tension) to 0.90 (anger) for the “past week” response timeframe.

### 2.3. Procedure

Data collection occurred from April to August 2023. This was a time during which athletes were involved in preparation for national and international competitions in Brazil. All athletes involved in the CUIDAR program were eligible to participate in the study. Mood assessment using the BRAMS measure occurred using a Google Forms online questionnaire. The athletes accessed the questionnaire on their mobile phones while being supervised by team coaching staff or strength and conditioning experts, who were trained in how to complete the BRAMS correctly. Data collection occurred in the athletes’ usual training environment. Mood using the “how are you feeling right now” timeframe was assessed before or after the first training session of the week, whereas mood using the “how have you been feeling during the past week” was assessed at the end of the week before or after the last training session. Data collection occurred between one and three weeks prior to the participants’ first competition of the season. Prior to data collection, all athletes provided informed consent and were notified that their participation was voluntary and could be withdrawn at any time. The young athletes signed the informed consent along with the signature of their parents/guardians. Ethical approval for the study to be conducted was provided by the University of Southern Queensland, Human Research Ethics Committee (#ETH2023-0046). 

### 2.4. Data Analysis

Data were analysed using IBM (USA) SPSS version 29 [66]. To assist with the interpretation of mood subscale scores, all raw subscale scores were transformed with reference to tables of normative data for athletes [46] into standard scores (T-scores), which have a mean of 50 and a standard deviation of 10. Following data screening, a MANOVA was conducted to compare the mood scores reported using the “right now” response timeframe with those reported using the “past week” response timeframe. Then, a seeded K-means cluster analysis was used to investigate whether the six mood profile clusters previously identified in the literature, referred to as the iceberg, surface, submerged, shark fin, inverse iceberg, and inverse Everest profiles [45,46,53,54,55,56,57,58], were also evident among the Brazilian sample. K-means cluster analysis is the recommended procedure to be used when there is prior knowledge of hypothesized clusters, as was the case in the present study [67,68]. To assess external validity [69], the emergent clusters were compared visually to those in Figure 1 [46] and elsewhere in the literature [45,53,54,55,56,57,58]. Next, the robustness of the cluster structures in our sample was assessed using discriminant function analysis and, finally, chi-squared analyses were used to identify if cluster prevalence varied significantly by response timeframe (right now/past week), sex (female/male), age group (12–17 years/18+ years), and social vulnerability (vulnerable/not vulnerable).

## 3. Results

### 3.1. Data Screening

As the BRAMS was completed by participants via an online questionnaire, no missing or out-of-range responses were recorded. In terms of univariate normality, the BRAMS subscales of anger, confusion, and depression showed a non-normal distribution of scores in both the “right now” and “past week” groups. Non-normal distributions are common for negative mood dimensions, which typically show a low proportion of high scores and a much higher proportion of low scores [20,21,45,46]. Those who report high scores on negatively-valenced mood subscales are seen as having elevated risk of mental health issues and therefore are of particular interest to applied practitioners and should be retained in the dataset where possible. Moreover, it is recommended [70] that interval-level data from self-report measures, as is the case with the BRAMS, should not be transformed and this recommendation was followed. In terms of multivariate normality, 46 multivariate outliers were identified using Mahalanobis distances at *p* < 0.001 in the “right now” group and another 37 in the “past week” group. Each of these cases was scrutinized individually for response bias, such as straight line, extreme, or acquiescent responding [71,72], but none was identified.

### 3.2. “Right Now” vs. “Past Week” Mood Scores

The results of a MANOVA to compare mean T-scores for “right now” and “past week” response time frames are shown in Table 2. Mean scores for the “past week” response timeframe were higher than the “right now” response timeframe for all mood subscales except vigor. The multivariate statistic was significant (Hotelling’s T = 0.091, F (6, 891) = 13.48, *p* < 0.001, η^2^_p_ = 0.083), indicating that 8.3% of the variance in mood scores was accounted for by the response timeframe. Follow-up univariate analyses showed that “past week” fatigue scores were significantly higher than “right now” scores (d = 0.56, moderate effect), as were depression scores (d = 0.19, small effect), and tension scores (d = 0.18, small effect). As the MANOVA procedure compared six mood subscales simultaneously, a Bonferroni adjustment was made to the alpha level, resulting in the use of *p* < 0.008 rather than *p* < 0.05 (0.05 ÷ 6 = 0.008) to signify a significant difference [73].

### 3.3. Cluster Analysis

**Figure 2:**
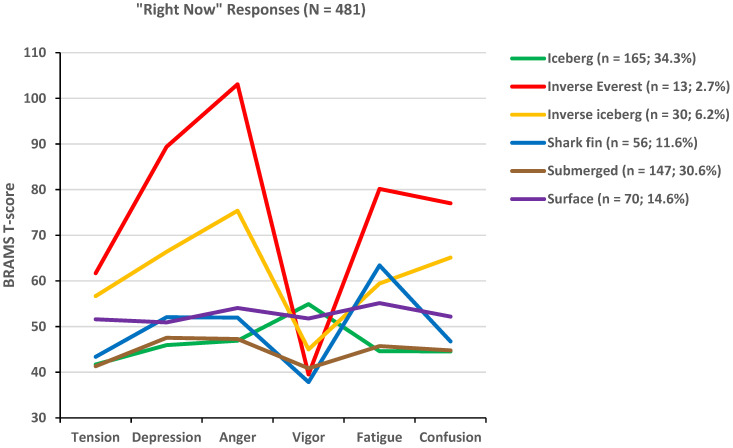
Graphical representation of six mood profile clusters identified in the “right now” group.

**Figure 3:**
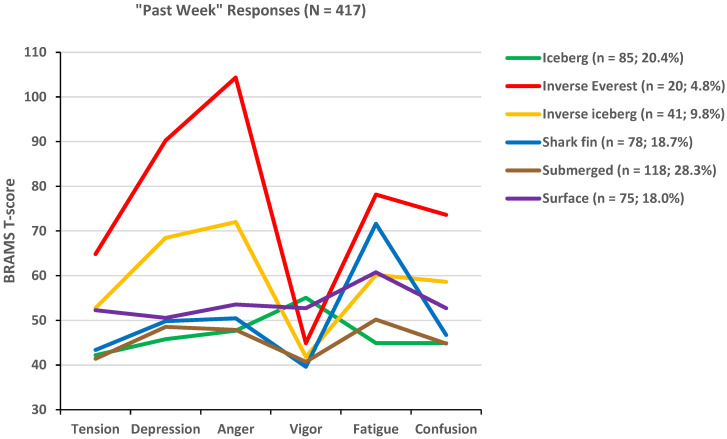
Graphical representation of six mood profile clusters identified in the “past week” group.

The cluster analysis results are shown graphically in Figure 2 (“right now” group) and Figure 3 (“past week” group). The six clusters identified in the literature, the iceberg, surface, submerged, shark fin, inverse iceberg, and inverse Everest profiles, were clearly identified in both response timeframe groups. In the “right now” group (Figure 2), the iceberg profile, which is the most positive of the six profiles and is associated with good mental health and superior athletic performance, was the most prevalent mood cluster (34.3%). The least prevalent profiles in the “right now” group were the more negative profiles, the inverse Everest (2.7%), inverse iceberg (6.2%), and shark fin profiles (11.6%), all of which are associated with increased risk of mental health issues and underperformance in sport.

In the “past week” group (Figure 3), the submerged profile was the most prevalent profile (28.3%), followed by the iceberg profile (20.4%), whereas the least prevalent profiles were the inverse Everest (4.8%) and inverse iceberg profiles (9.8%), with similar prevalence for the shark fin (18.7%) and surface profiles (18.0%). Descriptive statistics for the 6-cluster solutions are shown in Table 3 (“right now” group) and Table 4 (“past week” group).

### 3.4. Cluster Strength

Cluster strength was assessed using a discriminant function analysis, the results of which are shown in Table 5. For the “right now” group, 92.9% of cases were re-classified into their original groupings, with the first three functions accounting for 97.7% of the cumulative variance (i.e., 73.8%, 19.0%, and 4.9%, respectively). The Wilks’ Lambda test was significant (*p* < 0.001), signalling that each function had high discriminatory power. For the “past week” group, a somewhat lower proportion of cases, 89.0%, were re-classified into their original groupings. In this instance, the first three functions accounted for 98.5% of the cumulative variance (i.e., 62.0%, 24.5%, and 12.1%, respectively). The Wilks’ Lambda test was significant (*p* < 0.001), again indicating high discriminatory power for each function.

**Figure 4:**
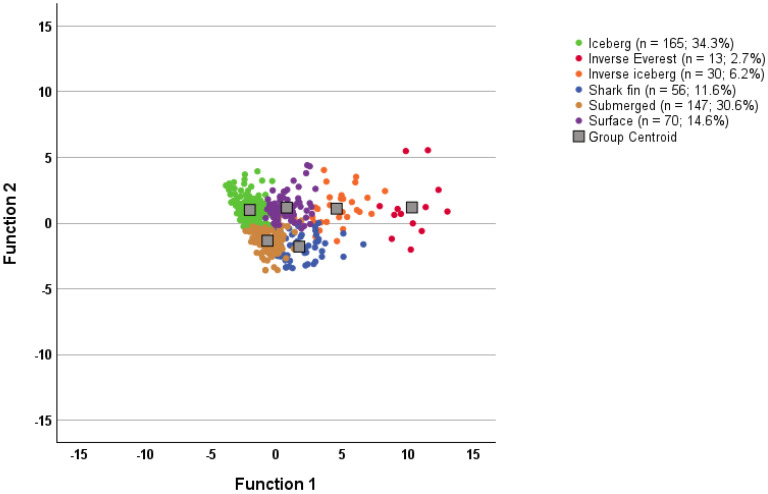
Canonical discriminant functions for the “right now” group (n = 481).

**Figure 5:**
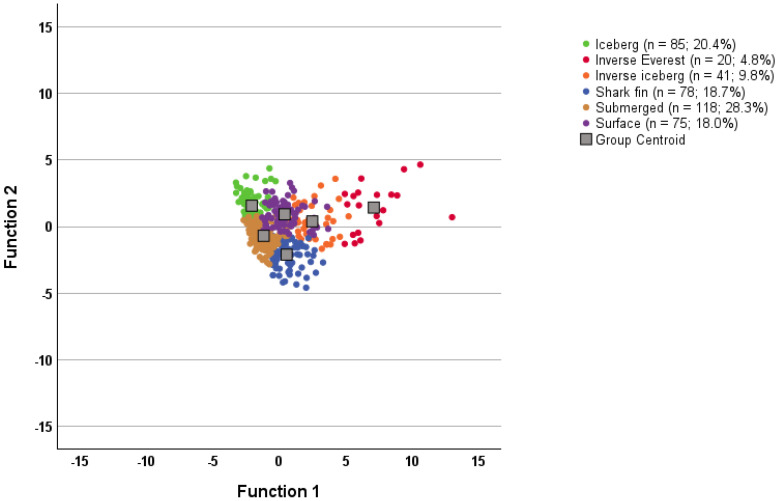
Canonical discriminant functions for the “past week” group (n = 417).

Canonical discriminant plots (see Figure 4 and Figure 5) show the compression of cases around the group centroids. The more positive profiles, namely the iceberg, submerged, and surface profiles, showed the greatest compaction for both response timeframes. Collectively, the discrimination function analysis results point to the cluster solutions being a good fit for the data for both groups.

### 3.5. Between-Group Differences in Cluster Prevalence

A series of chi-squared analyses were used to investigate if the prevalence of specific mood profile clusters varied by the demographic variables of interest (sex, age group, social vulnerability). The results are shown in Table 6. Significant between-group differences were identified from adjusted residual values, with critical values of ±1.96, ±2.58, and ±3.29 indicating significant differences at *p* < 0.05, *p* < 0.01, and *p* < 0.001, respectively [74]. The overall distribution of clusters varied significantly by sex (*p* < 0.001) for both the “right now” and “past week” groups and by social vulnerability (*p* < 0.05) for the “past week” group. The overall chi-squared value for the age group was not significant.

Considering the prevalence of specific clusters by sex, compared to females, males were over-represented in the iceberg profile in both timeframes (*p* < 0.001) and under-represented in the inverse Everest (*p* < 0.05), shark fin (*p* < 0.05), and submerged (*p* < 0.001) profiles in the “right now” group. In the “past week” group, compared to females, males were under-represented in the inverse iceberg (*p* < 0.01) and submerged (*p* < 0.05) profiles.

For age, compared to the 18+ years group, those in the ≤ 17 years group were over-represented in the iceberg profile (*p* < 0.05) for both timeframes. Further, although not reaching significance, there was a clear trend in the data for the 18+ years group to be over-represented in the clusters associated with greater risk of mental health issues, namely, the inverse Everest, inverse iceberg, and shark fin profiles. 

With regard to social vulnerability, those who were classified as vulnerable were over-represented in the submerged profile (*p* < 0.05) and under-represented in the surface profile (*p* < 0.05) in the “right now” group. In the “past week” group, participants classified as socially vulnerable were over-represented in the submerged profile (*p* < 0.01) compared to those classified as not socially vulnerable.

## 4. Discussion

This paper reports on an investigation of youth and elite athletes from a sports club in Rio de Janeiro, Brazil, participating in a multidisciplinary support program known as CUIDAR. The program is designed to promote health and improve the athletes’ sporting performance through the use of evidence-based strategies. Each area of support operates according to the stage of maturation and career of the athletes. In youth teams, the focus is on factors that contribute to the athletes’ long-term holistic and sporting growth, such as coordination skills, injury prevention programs, and educational lectures on topics such as nutrition, sleep, and mental health. Adult athletes, many of whom are professional athletes, receive individualized physical preparation and nutritional plans, injury prevention programs, and recovery strategies, with the aim of optimizing performance and safeguarding health. Mood profiling using the Brazil Mood Scale (BRAMS) occurs regularly with all athletes in the program to help inform support staff about how athletes are coping with training demands and to monitor athletes for their wellbeing status and risks to mental health. Both the “right now” and “past week” response timeframes are used.

The primary aim of the present study was to test whether six mood profile clusters identified in the literature [45,46,53,54,55,56,57,58], referred to as the iceberg, inverse iceberg, inverse Everest, surface, submerged, and shark fin profiles, would also be found among youth and elite athletes who were part of the CUIDAR program. Researchers have proposed that the temporal reference of “right now” is particularly sensitive to situational influences, making it especially valuable in evaluating the relationship between mood states and impending performance in sports settings [20,44]. Conversely, mood evaluations related to weekly periods using the “past week” reference period tend to capture more enduring feelings rather than short-term emotional responses [15,44]. Both response timeframes have their place in high-performance sports environments. Assessments of mood taken using the “past week” reference period towards the end of a training micro-cycle facilitate the monitoring of mental health status [46] and provide insights into subsequent athletic performance [11], enabling adjustments to training demands to be implemented in the subsequent micro-cycle.

Consistent with H1, a seeded k-means cluster analysis identified six mood clusters previously found in English-speaking samples [45,46], and among speakers of Brazilian Portuguese [53], Chinese [54], Greek [58], Italian [55], and Lithuanian [56]. Clusters were evident for both the “right now” and “past week” groups. The clusters were robust, and cases were classified with a high level of accuracy (92.9% for “right now” and 89.0% for “past week” groups) into their predicted clusters, showing an appropriate level of reliability for a high-performance sports environment. A substantial proportion of participants (79.5% for “right now” and 66.7% for “past week”) reported positive (iceberg) or neutral (submerged and surface) mood profiles, suggestive of good performance and positive mental health. The mood profiles linked to an elevated risk of mental ill-health, sports injuries, and reduced performance—the inverse Everest, inverse iceberg, and shark fin profiles—were clearly identified, although collectively reported by only a small minority of athletes (20.5% for “right now” and 33.3% for “past week”). Compared to the normative cluster prevalence for “right now” responses, which were based on a sample of nearly 16,000 participants [46], the sample of Brazilian athletes reported fewer negative profiles (20.5% vs. 31.9%), and more positive (34.3% vs. 28.5%) and neutral (45.2% vs. 39.5%) profiles. This trend towards more positive and less negative mood profiles among Brazilian athletes compared to the normative sample was also found by Brandão et al. [53] among a sample of 953 young Brazilian athletes. There are several potential explanations for the low prevalence of negative mood profiles in Brazilian athletic populations. Firstly, it may be reflective of a more general low prevalence of risk to mental health in Brazil, although this appears unlikely given that the country ranked 5th highest globally in 2024 for depression prevalence [74] and had an overall prevalence of mental health disorders during the 2015–2019 period of 16.7% [75], well above the global average of 12.5% [76]. It should be noted, however, that involvement in sports and physical activity more generally, affords some level of protection against mental ill-health [77,78], explaining why athletes would be expected to report more positive moods than the general population [46]. A second explanation is that the low prevalence of negative mood profiles is associated with the relatively young age of the participants in the present study and those recruited by Brandão et al. [53]. The Global Burden of Disease study results [79] show that the prevalence of mental health disorders in Brazil is lower among adolescents than adults. A third plausible explanation for our findings is that because all participants in the study were being supported by health professionals within the CUIDAR program, this was having a positive influence and lowering their risk of mental health issues. 

Several differences in cluster prevalence were identified between groups. As hypothesized (H2), significant sex and age differences were found for both response timeframes, with males and youth athletes reporting a higher prevalence of positive (iceberg) mood profiles than females and adult athletes. Additionally, female athletes showed a higher prevalence of negative (inverse Everest and shark fin) mood profiles than male athletes in the “right now” group and a higher prevalence of the inverse iceberg profile in the “past week” group. The observed trend of males reporting more positive mood profiles than females is consistent with the results of several previous investigations using the same or similar methodology [53,54,55,56,57,58] and was also reported in the original clusters paper by Parsons-Smith et al. [45], as well as the most recent norms paper [46]. This trend is also consistent with the fact that females experience mood disorders at nearly twice the rate of males [80,81], including in Brazil [75,79]. Explanations for this sex difference relate to biological factors, particularly the influence of sex hormones and differing reactions to stress [81,82,83], to psychological factors, notably lower self-esteem and higher rates of body shame and rumination [84], and to societal factors, such as gender inequality and discrimination [84,85,86].

Age differences were limited to the significantly higher prevalence of iceberg profiles among participants aged up to 17 years compared to those aged 18 years and over, for both response timeframes. Comparison with previous findings is difficult because the present study is the first study to have compared mood profile clusters of youth and adult athletes, and the first study to have used the BRAMS “past week” response timeframe with an athlete population. Previous investigations of mood profile clusters have typically shown a greater prevalence of positive mood profiles and a lesser prevalence of negative mood profiles among older adult groups compared to younger adult groups [56,57,58]. However, data from Brazil show a clear increase in the prevalence of mental health issues as people move from late adolescence into the early adult years [79]. Given that more than 60% of our sample were adolescents and many of the adult athletes were still young adults in the 18–25 age range, which is the peak age for the onset of mood disorders globally [87], then our results related to age can be seen to be consistent with the extant literature.

Regarding social vulnerability, and counter to our hypothesis (H3), no differences in prevalence emerged for positive (iceberg) and negative (inverse Everest, inverse iceberg, shark fin) profiles between participants who were classified as vulnerable and those who were classified as not vulnerable. The only differences that occurred related to the more neutral profiles, with the vulnerable group having a higher prevalence of submerged profiles for both “right now” and “past week” groups and a lower prevalence of the surface profile for the “right now” group. Given the existing evidence that socially vulnerable individuals are more prone to mental health issues [62,63] coupled with the findings that individuals living in the favelas of Rio de Janeiro, who are exposed to violence and extreme social disadvantage, are especially vulnerable to psychological distress [64], the absence of differences related to negative mood profiles was unexpected. By way of explanation, it is plausible that the social support provided to the vulnerable athletes in the CUIDAR program, many of whom lived in the favelas, by teammates and coaches plus the support of health and medical professionals, helped to build resilience [88,89] and provided some sort of protective effect to safeguard against mental health issues. Another explanation is grounded in the recent evidence provided by Renton and Guilherme [90] showing the positive impact of community-based sports programs on the mental health of people living in areas of extreme social disadvantage in Brazil. Renton and Guilherme explained the mental health benefits of sports participation by the effects of physical activity, social ties, community participation, and empowerment, which combined to build resilience amid difficult socioeconomic circumstances [90].

Consensus statements issued by the International Olympic Committee [2,91] and the International Society of Sport Psychology [1] emphasize that sports organizations have a solemn responsibility to protect the physical and mental health of athletes. This responsibility underpins the ethos of the CUIDAR program and its use of regular mood profiling with athletes. The present study reflects an ongoing commitment to base the support provided by medical and health professionals at the club on the latest scientific knowledge, in line with the best practice principles of knowledge transfer [92,93]. A recent framework proposed by Lundqvist and colleagues [94] emphasized the need to align intervention types with the nature and severity of mental health conditions. In particular, the framework emphasizes that low-level athlete wellbeing issues related to life and sports satisfaction can be addressed via the provision of socially supportive environments and mental skills training delivered by coaches and mental health professionals, whereas more serious pre-clinical issues and psychiatric disorders require support from those with clinical psychology expertise and certified healthcare professionals, respectively. This is indeed the approach embraced by the CUIDAR team, with mood profiling used to identify those individual athletes needing more intensive support. 

In summary, the observed prevalence rates suggest a relatively low risk of mental ill-health among the sample of Brazilian young and elite athletes, regardless of sex, age, or level of social vulnerability. Despite the low risk overall, about 1 in 5 of the participants reported a “right now” mood profile suggestive of an elevated risk of mental health issues. Within the CUIDAR screening program, such individuals are flagged for follow-up by a qualified mental health professional (i.e., psychologist, nurse, or medico), underlining the value of regular mood profiling or use of other types of psychological screening methods among large numbers of athletes involved in intense training [49]. Further, the present study was among the first to use the BRAMS “past week” response timeframe to assess mood responses among athletes, with about 1 in 3 participants reporting a profile associated with risk of mental ill-health. It was established previously [95] that scores on mood subscales tend to be higher when using the “past week” response timeframe compared to the mean of multiple “right now” profiles covering the same time period, which is consistent with the present results. This has been explained by the tendency for recollections of the intensity of emotional responses to be exaggerated when ‘‘over time’’ summaries are reported by participants [96]. Regarding the relative advantages and disadvantages of the two response timeframes, the “past week” reference period lends itself to function as an effective tool to monitor mental health status and risk of mental ill-health [46,47], whereas the “right now” reference period is more suited to providing insights into impending performance outcomes [95]. 

As a result of the observed mood differences associated with the two reference periods, separate tables of normative data specific to the response timeframe being used, are recommended. Given that “right now” norms already exist for the BRAMS, the generation of norms based on a “past week” response timeframe is a suitable topic for future research in a Brazilian context. Moreover, given evidence that the English-language version of the Brunel Mood Scale has demonstrated utility in screening individuals for risk of clinical conditions, including eating disorders [97,98], post-traumatic stress [99], and suicide ideation [100], as well as risk of athletic injury [48], there is considerable scope for further research using the BRAMS to investigate the antecedents and behavioural consequences of the six mood profile clusters among Brazilian athlete and non-athlete populations.

### 4.1. Limitations

Limitations of the present study are acknowledged. Firstly, all self-report data include inherent limitations, including distortion of responses due to social desirability or reluctance to reveal personal feelings [20,65], although there is no reason to assume that the present study was affected in this way to a greater extent than any other study using self-report data. Secondly, to prevent potential bias, data collection responsibilities were delegated to CUIDAR support team members because the first author held a managerial position within the organization. Although all individuals involved in the collection of data were trained in administering the BRAMS, the influence of having data collected by different individuals is unknown and could be considered a limitation.

### 4.2. Conclusions 

The six mood clusters previously identified in the literature were identified in youth and elite-level Brazilian athletes using both “right now” and “past week” versions of the BRAMS. Comparisons between sex and age groups revealed that males and youth athletes reported a higher prevalence of positive (iceberg) mood profiles than females and adult athletes, respectively, using both timeframes. Finally, no differences in prevalence emerged for positive and negative profiles between participants classified as socially vulnerable and those classified as not vulnerable, though the vulnerable group showed a higher prevalence of submerged profiles in both timeframes and a lower prevalence of the surface profile in the “right now” timeframe. 

### 4.3. Practical Applications

These findings highlight the importance of personalized support and interventions to respond to mental health needs based on individual characteristics and circumstances. The commitment to using evidence-based strategies and regular mood profiles to monitor the mental health status of the athletes aligns with the broader responsibility of sports organizations to safeguard athletes’ physical and mental wellbeing. The results highlight the potential benefits of integrating mental health screening and support programs into sports clubs to promote athlete wellbeing and optimize performance. Finally, the present study sheds light on the mental health outlook of Brazilian athletes and emphasizes the importance of proactive mental health monitoring and support in high-performance sporting environments. 

## Figures and Tables

**Table 1:** Situational and demographic characteristics of 898 participants [43].

Source	Group	Right Now		Past Week	
		n	%	n	%
Sex	Male	282	58.6	229	54.9
	Female	199	41.4	188	45.1
Age Group	12–17 years	303	63.0	252	60.4
	18+ years	178	37.0	165	39.6
Social Vulnerability	Vulnerable	258	55.6	232	57.9
	Not vulnerable	206	44.4	169	42.1
Sport	Artistic Swimming	27	5.6	24	5.8
	Basketball	55	11.4	22	5.3
	Gymnastics	10	2.1	10	2.4
	Judo	40	8.3	35	8.4
	Rowing	104	21.6	98	23.5
	Swimming	75	15.6	70	16.8
	Volleyball	93	19.3	83	19.9
	Water Polo	77	16.0	75	18.0
Total	All	481	100.0	417	100.0

Note. For 33 participants, social vulnerability status could not be determined.

**Table 2:** Results of a MANOVA to compare BRAMS T-scores by response time frame.

	Right Now (n = 481)		Past Week (n = 417)		*F*
	*M*	*SD*	*M*	*SD*	
Tension	44.69	7.34	46.13	8.34	7.59 *
Depression	50.31	10.54	52.53	13.40	7.74 *
Anger	51.94	14.13	54.42	16.51	5.90
Vigor	47.14	8.62	45.90	8.50	4.68
Fatigue	50.55	10.43	57.34	12.76	76.97 ^†^
Confusion	48.15	10.11	49.35	9.69	3.26

Note. Hotelling’s T = 0.091, F (6, 891) = 13.48 ^†^, η^2^_p_ = 0.083. ^†^ *p* < 0.001, * *p* < 0.008.

**Table 3:** Descriptive statistics of the 6-cluster solution for the “right now” group (n = 481).

**Source**	**Iceberg (n = 165; 34.3%)**			**Inverse Everest (n = 13; 2.7%)**			**Inverse Iceberg (n = 30; 6.2%)**		
	***M***	***SD***	**95% CI**	***M***	***SD***	**95% CI**	***M***	***SD***	**95% CI**
Tension	41.69	3.76	(41.11, 42.27)	61.69	7.60	(57.10, 66.28)	56.67	8.92	(53.33, 60.00)
Depression	45.93	2.45	(45.55, 46.30)	89.38	18.89	(77.97, 100.80)	66.37	13.76	(61.23, 71.50)
Anger	46.90	4.33	(46.23, 47.56)	103.08	23.70	(88.76, 117.40)	75.40	21.62	(67.33, 83.47)
Vigor	54.93	5.35	(54.10, 55.75)	39.54	9.39	(33.87, 45.21)	45.00	6.20	(42.69, 47.31)
Fatigue	44.61	4.57	(43.90, 45.31)	80.15	11.15	(73.42, 86.89)	59.43	8.52	(56.25, 62.61)
Confusion	44.56	3.56	(44.01, 45.10)	77.00	27.16	(60.59, 93.41)	65.10	14.46	(59.70, 70.50)
**Source**	**Shark Fin (n = 56; 11.6%)**			**Submerged (n = 147; 30.6%)**			**Surface (n = 70; 14.6%)**		
	***M***	***SD***	**95% CI**	***M***	***SD***	**95% CI**	***M***	***SD***	**95% CI**
Tension	43.38	4.96	(42.05, 44.70)	41.31	3.54	(40.73, 41.88)	51.60	6.25	(50.11, 53.09)
Depression	52.04	8.27	(49.82, 54.25)	47.54	4.41	(46.83, 48.26)	50.93	7.57	(49.12, 52.73)
Anger	51.96	9.15	(49.51 54.42)	47.27	5.52	(46.37, 48.17)	54.07	8.66	(52.01, 56.14)
Vigor	37.84	5.44	(36.38, 39.30)	40.86	4.15	(40.19, 41.54)	51.76	4.82	(50.61, 52.91)
Fatigue	63.41	9.53	(60.86, 65.96)	45.71	4.32	(45.00, 46.41)	55.14	7.34	(53.39, 56.89)
Confusion	46.75	5.82	(45.19, 48.31)	44.78	3.53	(44.20, 45.35)	52.19	7.48	(50.40, 53.97)

**Table 4:** Descriptive statistics of the 6-cluster solution for the “past week” group (n = 417).

**Source**	**Iceberg (n = 85; 20.4%)**			**Inverse Everest (n = 20; 4.8%)**			**Inverse Iceberg (n = 41; 9.8%)**		
	***M***	***SD***	**95% CI**	***M***	***SD***	**95% CI**	***M***	***SD***	**95% CI**
Tension	42.21	4.31	(41.28, 43.14)	64.85	9.86	(60.23, 69.47)	52.76	5.24	(51.10, 54.41)
Depression	45.78	2.64	(45.21, 46.35)	90.25	21.45	(80.21, 100.29)	68.46	13.95	(64.06, 72.87)
Anger	47.68	7.26	(46.12, 49.25)	104.35	23.80	(93.21, 115.49)	72.02	15.34	(67.18, 76.87)
Vigor	55.06	5.53	(53.87, 56.25)	44.90	7.48	(41.40, 48.40)	41.83	5.39	(40.13, 43.53)
Fatigue	44.91	4.62	(43.91, 45.90)	78.15	9.29	(73.80, 82.50)	60.17	10.26	(56.93, 63.41)
Confusion	44.88	4.25	(43.97, 45.80)	73.60	17.69	(65.32, 81.88)	58.63	7.61	(56.23, 61.04)
**Source**	**Shark Fin (n = 78; 18.7%)**			**Submerged (n = 118; 28.3%)**			**Surface (n = 75; 18.0%)**		
	***M***	***SD***	**95% CI**	***M***	***SD***	**95% CI**	***M***	***SD***	**95% CI**
Tension	43.36	5.66	(42.08, 44.64)	41.39	3.57	(40.74, 42.04)	52.28	7.56	(50.54, 54.02)
Depression	49.81	7.14	(48.20, 51.42)	48.54	6.34	(47.39, 49.70)	50.55	6.85	(48.97, 52.12)
Anger	50.46	7.54	(48.76, 52.16)	47.87	5.77	(46.82, 48.93)	53.55	10.23	(51.19, 55.90)
Vigor	39.63	5.55	(38.38, 40.88)	40.71	5.09	(39.78, 41.64)	52.72	5.59	(51.43, 54.01)
Fatigue	71.64	7.77	(69.89, 73.39)	50.18	6.43	(49.01, 51.35)	60.75	8.69	(58.75, 62.75)
Confusion	46.73	5.41	(45.51, 47.95)	44.81	4.13	(44.05, 45.56)	52.72	7.57	(50.98, 54.46)

**Table 5:** Classification of discriminant functions for “right now” (n = 481) and “past week” (n = 417) groups.

Cluster	Predicted Group Membership						n	%
	1	2	3	4	5	6		
Right Now								
1	158	0	0	0	1	6	165	95.8
2	0	13	0	0	0	0	13	100.0
3	0	0	28	1	0	1	30	93.3
4	0	0	2	47	5	2	56	83.9
5	8	0	1	1	134	3	147	91.2
6	2	0	1	0	0	67	70	95.7
Past Week								
1	76	0	0	0	4	5	85	89.4
2	0	20	0	0	0	0	20	100.0
3	0	2	34	2	0	3	41	82.9
4	0	0	2	70	4	2	78	89.7
5	9	0	4	1	102	2	118	86.4
6	1	0	1	1	3	69	75	92.0

Note. 1 = Iceberg, 2 = Inverse Everest, 3 = Inverse iceberg, 4 = Shark fin, 5 = Submerged, 6 = Surface.

**Table 6:** Distribution of clusters by demographic variables (N = 898).

Source	Cluster											
	1	%	2	%	3	%	4	%	5	%	6	%
Right now (n = 481)												
Sex χ^2^(5) = 45.74^†^												
Male (n = 282)	126 ^†+^	44.7	4 *^−^	1.4	15	5.3	24 *^−^	8.5	66 ^†–^	23.4	47	16.7
Female (n = 199)	39 ^†–^	19.6	9 *^+^	4.5	15	7.5	32 *^+^	16.1	81 ^†+^	40.7	23	11.6
Age group χ^2^(5) = 8.33												
≤17 years (n = 303)	114 *^+^	37.6	7	2.3	15	5.0	30	9.9	96	31.7	41	13.5
18+ years (n = 178)	51 *^−^	28.7	6	3.4	15	8.4	26	14.6	51	28.7	29	16.3
Vulnerability χ^2^(5) = 10.67												
No (n = 206)	76	36.9	5	2.4	11	5.3	24	11.7	50 *^−^	24.3	40 *^+^	19.4
Yes (n = 258)	83	32.2	8	3.1	17	6.6	31	12.0	90 *^+^	34.9	29 *^−^	11.2
Past week (n = 417)												
Sex χ^2^(5) = 33.43^†^												
Male (n = 229)	66 ^†+^	28.8	10	4.4	13 ^§–^	5.7	39	17.0	54 *^−^	23.6	47	20.5
Female (n = 188)	19 ^†–^	10.1	10	5.3	28 ^§+^	14.9	39	20.7	64 *^+^	34.0	28	14.9
Age group χ^2^(5) = 8.65												
≤17 years (n = 252)	61 *^+^	24.2	9	3.6	22	8.7	45	17.9	67	26.6	48	19.0
18+ years (n = 165)	24 *^−^	14.5	11	6.7	19	11.5	33	20.0	51	30.9	27	16.4
Vulnerability χ^2^(5) = 11.1 *												
No (n = 169)	36	21.3	10	5.9	15	8.9	35	20.7	35 ^§–^	20.7	38	22.5
Yes (n = 232)	42	18.1	10	4.3	24	10.3	41	17.7	80 ^§+^	34.5	35	15.1

Note. 1 = Iceberg, 2 = Inverse Everest, 3 = Inverse iceberg, 4 = Shark fin, 5 = Submerged, 6 = Surface; ^+^ = over-represented, ^−^ = under-represented; ^†^ *p* < 0.001; ^§^ *p* < 0.01; * *p* < 0.05.

## Data Availability

Restrictions apply to the availability of these data. Data were obtained from Clube de Regatas do Flamengo, Rio de Janeiro, Brazil, and are available from the corresponding author with the permission of Clube de Regatas do Flamengo.
